# *POU4F3* mutation screening in Japanese hearing loss patients: Massively parallel DNA sequencing-based analysis identified novel variants associated with autosomal dominant hearing loss

**DOI:** 10.1371/journal.pone.0177636

**Published:** 2017-05-17

**Authors:** Tomohiro Kitano, Maiko Miyagawa, Shin-ya Nishio, Hideaki Moteki, Kiyoshi Oda, Kenji Ohyama, Hiromitsu Miyazaki, Hiroshi Hidaka, Ken-ichi Nakamura, Takaaki Murata, Rina Matsuoka, Yoko Ohta, Nobuhiro Nishiyama, Kozo Kumakawa, Sakiko Furutate, Satoshi Iwasaki, Takechiyo Yamada, Yumi Ohta, Natsumi Uehara, Yoshihiro Noguchi, Shin-ichi Usami

**Affiliations:** 1 Department of Otorhinolaryngology, Shinshu University School of Medicine, Matsumoto, Japan; 2 Department of Hearing Implant Sciences, Shinshu University School of Medicine, Matsumoto, Japan; 3 Department of Otorhinolaryngology, Tohoku Rosai Hospital, Sendai, Japan; 4 Department of Otorhinolaryngology-Head and Neck Surgery, Tohoku University School of Medicine, Sendai, Japan; 5 Department of Otolaryngology-Head and Neck Surgery, Jichi Medical University School of Medicine, Shimotsuke, Japan; 6 Department of Otolaryngology-Head and Neck Surgery, Gunma University Graduate School of Medicine, Maebashi, Japan; 7 Department of Otorhinolaryngology, Juntendo University Faculty of Medicine, Tokyo, Japan; 8 Department of Otorhinolaryngology Head and Neck Surgery, Tokyo Medical University, Tokyo, Japan; 9 Department of Otorhinolaryngology, Toranomon Hospital, Tokyo, Japan; 10 Department of Otorhinolaryngology, International University of Health and Welfare, Mita Hospital, Tokyo, Japan; 11 Department of Otorhinolaryngology-Head and Neck Surgery, University of Fukui, Fukui, Japan; 12 Department of Otorhinolaryngology-Head and Neck Surgery, Osaka University Graduate School of Medicine, Osaka, Japan; 13 Department of Otolaryngology-Head and Neck Surgery, Kobe University School of Medicine, Kobe, Japan; Universidad de Salamanca, SPAIN

## Abstract

A variant in a transcription factor gene, *POU4F3*, is responsible for autosomal dominant nonsyndromic hereditary hearing loss, DFNA15. To date, 14 variants, including a whole deletion of *POU4F3*, have been reported to cause HL in various ethnic groups. In the present study, genetic screening for *POU4F3* variants was carried out for a large series of Japanese hearing loss (HL) patients to clarify the prevalence and clinical characteristics of DFNA15 in the Japanese population. Massively parallel DNA sequencing of 68 target candidate genes was utilized in 2,549 unrelated Japanese HL patients (probands) to identify genomic variations responsible for HL. The detailed clinical features in patients with *POU4F3* variants were collected from medical charts and analyzed. Novel 12 *POU4F3* likely pathogenic variants (six missense variants, three frameshift variants, and three nonsense variants) were successfully identified in 15 probands (2.5%) among 602 families exhibiting autosomal dominant HL, whereas no variants were detected in the other 1,947 probands with autosomal recessive or inheritance pattern unknown HL. To obtain the audiovestibular configuration of the patients harboring *POU4F3* variants, we collected audiograms and vestibular symptoms of the probands and their affected family members. Audiovestibular phenotypes in a total of 24 individuals from the 15 families possessing variants were characterized by progressive HL, with a large variation in the onset age and severity with or without vestibular symptoms observed. Pure-tone audiograms indicated the most prevalent configuration as mid-frequency HL type followed by high-frequency HL type, with asymmetry observed in approximately 20% of affected individuals. Analysis of the relationship between age and pure-tone average suggested that individuals with truncating variants showed earlier onset and slower progression of HL than did those with non-truncating variants. The present study showed that variants in *POU4F3* were a common cause of autosomal dominant HL.

## Introduction

Hearing loss (HL) is the most common sensory impairment, and is divided into prelingual HL (HL starts before speech development) and postlingual HL (HL occurs after speech development). It is estimated that at least two-thirds of prelingual HL patients exhibit hereditary HL. The inheritance patterns of this form of HL include autosomal recessive, autosomal dominant, X-linked, and mitochondrial. Autosomal dominant nonsyndromic hereditary HL (ADNSHL) is typically postlingual, and accounts for approximately 20% of nonsyndromic hereditary HL patients [1]. Thus far, 36 causative genes for ADNSHL have been identified [2].

One form of ADNSHL, DFNA15 (MIM# 602459), is caused by variants in the *POU4F3* gene, which was first identified in a large Israeli Jewish family [3]. *POU4F3* is located on chromosome 5q32, and encodes a POU family transcription factor: POU domain, class 4, transcription factor 3 (*POU4F3*). *POU4F3* consists of a 338 amino-acid polypeptide, and contains two DNA-binding domains in the form of a POU-specific domain and a Homeobox domain. In humans, *POU4F3* is not expressed in the brain, heart, placenta, skeletal muscle, lung, kidney, pancreas, or lymphoblast tissues, but is expressed in the fetal cochlea [3]. In mice, *Pou4f3* is uniquely and strongly expressed in the cochlear and vestibular hair cells of the inner ear [4, 5]. *Pou4f3* is reported to be essential for the final differentiation and survival of hair cells [6, 7]. Targeted deletion of both alleles of *Pou4f3* is responsible for profound deafness and balance impairment in mice because of complete cochlear and vestibular hair cell losses followed by a partial secondary loss of spiral and vestibular ganglion neurons [4, 5].

To date, 13 different variants in *POU4F3* [3, 8–18] and a whole deletion of *POU4F3* [19] have been reported to cause HL in various ethnic groups, including the Dutch, Japanese, Korean, Chinese, and Brazilian populations. Although previously reported papers have shown the clinical characteristics of patients with *POU4F3* variants, the detailed audiovestibular findings remain unknown [8, 9, 12, 20]. In the present study, we used massively parallel DNA sequencing (MPS) to detect pathogenic variants in *POU4F3* among a large series of Japanese HL patients. The aims of the study are to estimate the prevalence of *POU4F3* variants in the Japanese population with hereditary HL, and obtain a more precise description of the clinical features.

## Materials and methods

### Subjects

All procedures were approved by the Shinshu University Ethical Committee as well as the respective Ethical Committees of the other participating institutions, and were carried out after obtaining written informed consent from all subjects (or from their next of kin, caretaker, or guardian in the case of minors/children).

A total of 2,549 probands (age range: 0–79 years, mean age: 22 years) from unrelated Japanese HL families were enrolled from the 67 otolaryngology departments across Japan participating in the present study among May 2012 to September 2016. The probands’ ages ranged from 0 to 79 years (mean ± SD: 22.1 ± 19.7). The hereditary patterns of the HL in the probands’ families were autosomal dominant in 602, autosomal recessive in 1,577, and unknown inheritance mode in 370.

### Variant analysis

Amplicon libraries were prepared using an Ion AmpliSeq^™^ Custom Panel (Applied Biosystems, Life Technologies), in accordance with the manufacturer’s instructions, for 68 genes reported to cause non-syndromic hereditary HL (S1 Table). The detailed protocol has been described elsewhere [21]. After preparation, the amplicon libraries were diluted to 20pM and equal amounts of 6 libraries for 6 patients were pooled for one sequence reaction.

Emulsion PCR and sequencing were performed according to the manufacturer’s instructions. The detailed protocol has been described elsewhere [21]. MPS was performed with an Ion Torrent Personal Genome Machine (PGM) system using an Ion PGM^™^ 200 Sequencing Kit and an Ion 318^™^ Chip (Life Technologies).

The sequence data were mapped against the human genome sequence (build GRCh37/hg19) with a Torrent Mapping Alignment Program. After sequence mapping, the DNA variant regions were piled up with Torrent Variant Caller plug-in software. After variant detection, their effects were analyzed using ANNOVAR software [22, 23]. The missense, nonsense, insertion/deletion and splicing variants were selected from among the identified variants. Variants were further selected as less than 1% of 1) the 1,000 genome database [24], 2) the 6,500 exome variants [25], 3) the Human Genetic Variation Database (dataset for 1,208 Japanese exome variants) [26], and 4) the 333 in-house Japanese normal hearing controls. Direct sequencing was utilized to confirm the selected variants.

The pathogenicity of a variant was evaluated by ACMG (American College of Medical Genetics) standards and guidelines [27]. For missense variants in particular, functional prediction software, including Sorting Intolerant from Tolerant (SIFT) [28], Polymorphism Phenotyping (PolyPhen2) [29], Likelihood Ratio Test (LRT) [30], Mutation Taster [31], Mutation Assessor [32], Functional Analysis through Hidden Markov Models (FATHMM) [33], Radial Support Vector Machine (RadialSVM), and Logistic Regression (LR) [34] were used on the wANNOVAR website. Conservation of the variantsite was also evaluated in 13 species from the Homologene website [35]. Segregation analysis was performed for each proband and their family members.

### Clinical evaluations

The onset age of HL, and the incidences of progressive HL and vertigo/dizziness were analyzed based on the medical charts of the 15 probands and nine their family members with *POU4F3* variants.

Pure-tone audiometry was performed to evaluate HL. Pure-tone average (PTA) was calculated from the audiometric thresholds at four frequencies (0.5, 1, 2, and 4 kHz). If an individual did not respond to the maximum hearing level at a frequency, 5 dB was added to the maximum hearing level. The severity of HL was assigned into mild (PTA: 20–40 dB HL), moderate (41–70 dB HL), severe (71–95 dB HL), and profound (>95 dB HL). Asymmetry in hearing was defined as a difference in PTA of > 10 dB between the right and left ears. The audiometric configurations were categorized to low-frequency, mid-frequency (U-shaped), high-frequency, flat, and deaf.

The findings of vestibular examinations, including caloric testing and the measurement of cervical vestibular evoked myogenic potentials (cVEMPs), were analyzed.

Intervention for HL, including the use of hearing aids or cochlear implants, was investigated based on the medical charts.

## Results

### Detected variants and the pathogenicity

A total of 12 possibly disease-causing variants were detected in 15 (2.5%) of 602 probands with autosomal dominant HL (Table 1), whereas no pathogenic variants were found in the other 1,947 probands with autosomal recessive or inheritance pattern unknown HL. No variants in the other 67 deafness genes were identified in the 15 probands. All variants were novel, and included six missense variants, three frameshift variants, and three nonsense variants. Among the 12 variants, eight were located in the POU-specific domain (amino acids 179–256) or Homeobox domain (amino acids 272–332). Of the other four variants, p.His25fs and p.Ile123fs were predicted to show premature translation stops at codons 40 and 127, respectively. Therefore, these two frameshift variants and a nonsense variant (p.Gln143Ter), presumably resulted in the absence of the two domains.

**Table 1:** Possible causative variants found in this study.

				Prediction Score*										
Nucleotide Change	Exon	Amino Acid Change	Domain	SIFT	PolyPhen2_HDIV	PolyPhen2_HVAR	LRT	Mut_Taster	Mut_Assessor	FATHMM	RadialSVM	LR	Evolutional Conservation	Allele Frequency in Controls***
c.74dupA	1	p.His25fs												0
c.191A>T	2	p.Asp64Val		1.00	1.00	0.93	0.60	1.00	0.71	0.33	0.26	0.18	Yes	0
c.367delA	2	p.Ile123fs												0
c.427C>T	2	p.Gln143Ter												0
c.574G>T	2	p.Glu192Ter	POU-specific											0
c.581T>A	2	p.Phe194Tyr	POU-specific	0.99	1.00	1.00	1.00	1.00	0.76	0.48	0.55	0.82	Yes	0
c.665C>T	2	p.Ser222Leu	POU-specific	0.99	0.99	0.87	1.00	1.00	0.69	0.47	0.61	0.72	Yes**	0
c.680delC	2	p.Thr227fs	POU-specific											0
c.718A>T	2	p.Asn240Tyr	POU-specific	1.00	1.00	1.00	1.00	1.00	0.77	0.47	0.65	0.80	Yes	0
c.841A>G	2	p.Ile281Val	Homeobox	1.00	1.00	1.00	1.00	1.00	0.70	0.64	0.70	0.92	Yes	0
c.896C>T	2	p.Pro299Leu	Homeobox	1.00	1.00	1.00	1.00	1.00	0.61	0.56	0.71	0.92	Yes	0
c.976A>T	2	p.Arg326Ter	Homeobox											0

Abbreviation: SIFT, Sorting Intolerant from Tolerant; PolyPhen2_HDIV, Polymorphism Phenotyping v2 based on HumDIV; HVAR, HumVAR; LRT, Likelihood Ratio Test; Mut_Taster, Mutation Taster; Mut_Assessor, Mutation Assessor; FATHMM, Functional Analysis through Hidden Markov Models; RadialSVM, Radial Support Vector Machine; LR, Logistic Regression

* The prediction scores of each algorithm included on the wANNOVAR website were converted from the original scoring system. Scores closer to 1.0 indicated the variant was more damaging, and those closer to 0 indicated they were more tolerant.

** Amino acid (p.Ser222Leu) was conserved in mammalian species.

*** Allele frequency in 333 in-house controls (666 control alleles)

**Fig 1:**
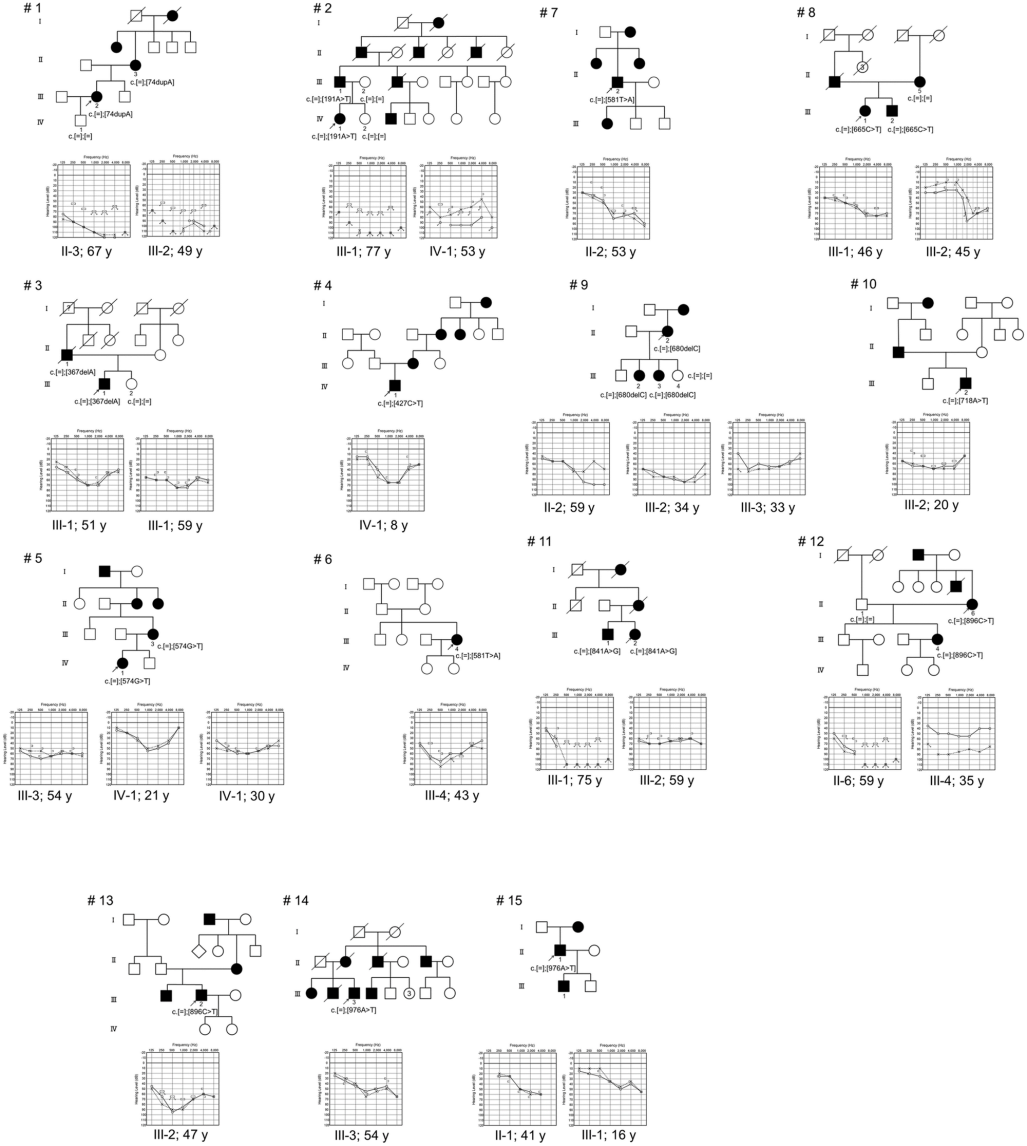
Pedigree and audiograms for each family with *POU4F3* variants. Arrow shows the probands in each family (family numbers #1-#15). Genetic findings for each individual tested are noted in the pedigree.

With respect to two variants (p.Phe194Tyr, p.Arg326Ter), genetic analysis was available only for the probands. However, these variants were detected in two unrelated probands (Table 2). The remaining eight variants were confirmed to segregate with HL in the respective families (Fig 1). All six missense variants were predicted to be pathogenic using the aforementioned software programs, and their corresponding amino acids were well conserved across species (Table 1). Further, none of the 12 variants was found in 333 in-house controls (666 control alleles). Taken together, the 12 variants were likely pathogenic.

**Table 2:** Clinical features of affected family members associated with *POU4F3* variants found in this study.

			HL			Pure-tone audiometry					
Family No.	Patient No.	Amino Acid Change	Onset	Progression	Vertigo/dizziness	Tested age (y)	PTA (R/L)	Severity (R/L)	Audiometric configuration (R/L)	Vestibular function (R/L)	Intervention
1	III-2	p.His25fs	20 y	Yes	No	49	112.5/110	profound/profound	HF/HF		CI
	II-3		20’s	Yes	No	67	105/106.3	profound/profound	DE/DE		HA
2	IV-1	p.Asp64Val	30’s	Yes	No	53	91.3/61.3	severe/moderate	MF/NC	abnormal/normal	HA
	III-1		10’s	Yes	No	77	115.0/115.0	profound/profound	DE/DE		HA
3	III-1	p.Ile123fs	40 y	Yes	No	51	62.5/58.8	moderate/moderate	MF/MF	normal/normal	HA
	II-1		40’s	Yes	N/A	N/A	N/A	N/A	N/A		HA
4	IV-1	p.Gln143Ter	3y	N/A	N/A	8	47.5/58.8	moderate/moderate	MF/MF		HA
5	III-3	p.Glu192Ter	30’s	Yes	No	54	63.8/58.8	moderate/moderate	MF/MF		N/A
	IV-1		17 y	Yes	No	21	45.0/41.3	moderate/moderate	MF/MF		N/A
6	III-4	p.Phe194Tyr	20 y	Yes	No	43	60.0/65.0	moderate/moderate	MF/MF		N/A
7	II-2	p.Phe194Tyr	10’s	Yes	No	53	67.5/67.5	moderate/moderate	HF/HF		HA
8	III-1	p.Ser222Leu	6 y	Yes	Yes	46	56.3/53.8	moderate/moderate	HF/HF		N/A
	III-2		6 y	Yes	No	45	43.8/23.8	moderate/mild	HF/HF		N/A
9	II-2	p.Thr227fs	N/A	Yes	No	59	80.0/65.0	severe/moderate	HF/MF		N/A
	III-2		infant	Yes	No	34	88.8/90.0	severe/severe	MF/MF		N/A
	III-3		infant	Yes	No	33	61.3/66.3	moderate/moderate	MF/MF		N/A
10	III-2	p.Asn240Tyr	6 y	Yes	Yes	20	66.3/68.8	moderate/moderate	MF/MF		HA
11	III-1	p.Ile281Val	54 y	Yes	No	59	67.5/70.0	moderate/moderate	HF/HF		HA
	III-2		50 y	Yes	No	75	115.0/115.0	profound/profound	DE/DE		CI*
12	II-6	p.Pro299Leu	27 y	Yes	Yes	59	106.3/110.0	profound/profound	HF/HF	normal/normal	CI
	III-4		26 y	Yes	Yes	35	50.0/85.0	moderate/severe	MF/flat		N/A
13	III-2	p.Pro299Leu	41 y	Yes	No	47	75.0/75.0	severe/severe	MF/MF		N/A
14	III-3	p.Arg326Ter	childhood	Yes	No	54	48.8/52.5	moderate/moderate	HF/HF		None
15	II-1	p.Arg326Ter	childhood	Yes	No	41	47.5/47.5	moderate/moderate	HF/HF		N/A

Abbreviations: y, year(s) old; R, right ear; L left ear; HL, hearing loss; DE, deaf; HF, high-frequency hearing loss; MF, mid-frequency hearing loss; NC: not classified; CI, cochlear implant; HA, hearing aid; N/A, not available

*preparing for CI surgery

### Clinical characteristics

Table 2 summarizes the clinical characteristics of 24 affected individuals from 15 families with *POU4F3* likely pathogenic variants. The onset age of HL varied markedly from 3 to 54 years. No data on the progressive nature of the HL was available for one individual. The other 23 individuals had noticed a progression in HL. In addition, one individual (family 12: II-6) suffered from a rapid deterioration in hearing at some time during the course of HL (no audiogram was available). She received steroid therapy, but her hearing showed no recovery. With respect to vestibular symptoms, four individuals (16.7%) suffered from vertigo/dizziness.

Pure-tone audiograms were obtained in 23 individuals. All individuals exhibited bilateral moderate to profound HL. Asymmetric hearing was observed in five individuals (20.8%) (family 2: IV-1, 4: IV-1, 8: III-2, 9: II-2, and 12: III-4). Audiometric configuration included mid-frequency HL type in 21 ears, high-frequency HL type in 17 ears, flat type in one ear, and deaf in six ears. Audiometric configuration could not be classified in one ear.

**Fig 2:**
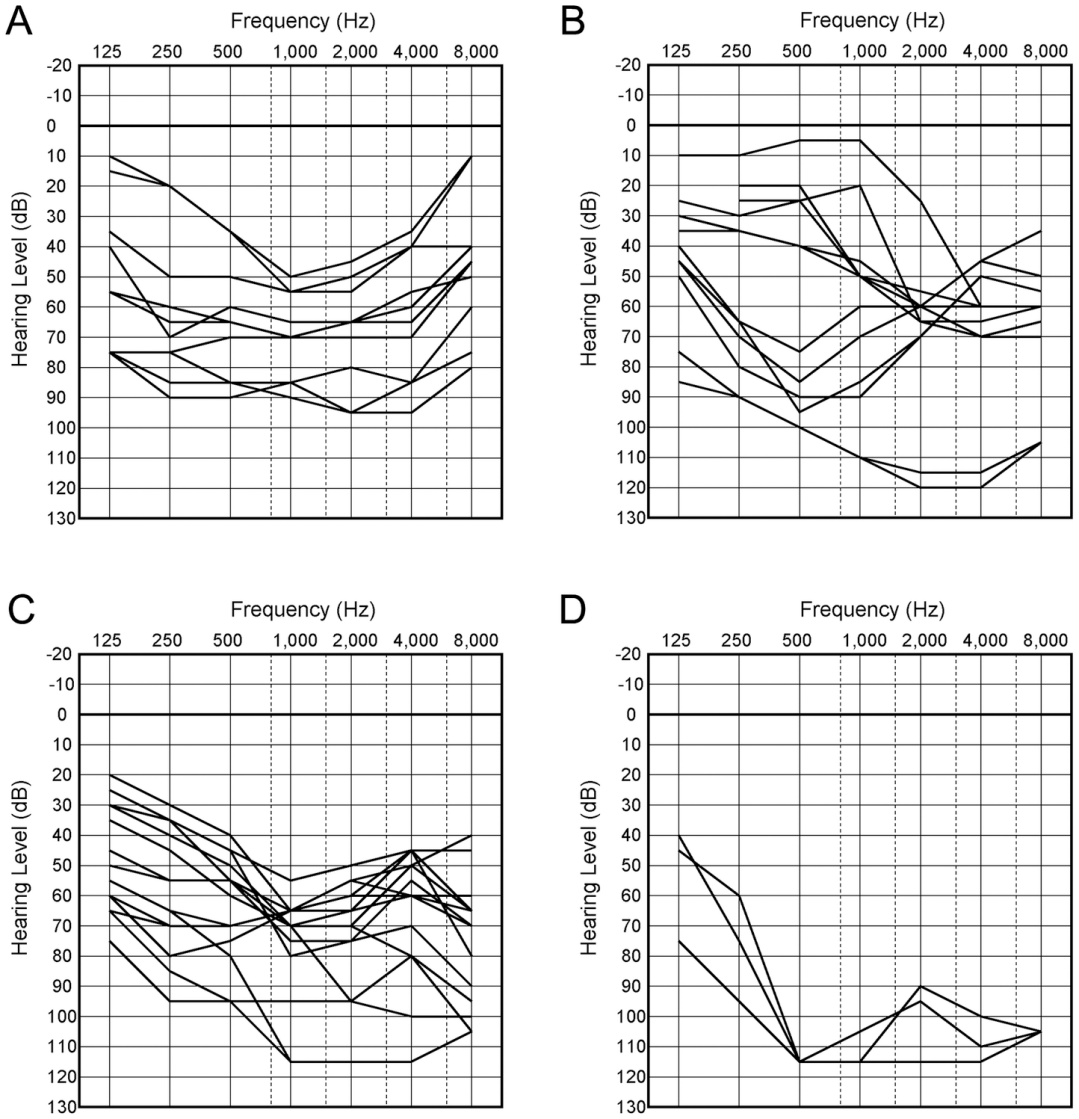
Overlapping audiograms in each age period. Pure-tone audiograms of both ears are shown by age period. (A) At age 20–39, all individuals have mid-frequency hearing loss. At age 40–49 (B) and age 50–59 (C), all ears have moderate to profound hearing loss at the frequencies of 4 and 8 kHz, and consequently some ears show high-frequency hearing loss. (D) At age 60 or older, all ears show profound HL with or without residual hearing at the lower frequencies.

Overlapping audiograms for each age period are shown in Fig 2. At age 20–39, all ears showed mid-frequency HL and some ears had normal or mild threshold elevation at 4 and 8 kHz. At age 40–59, all ears exhibited moderate to profound HL at the frequencies of 4 and 8 kHz, and consequently some ears showed high-frequency HL. At 60 or older, all ears showed profound HL, although some ears had residual hearing at the lower frequencies.

Serial pure-tone audiograms could be obtained for two unrelated individuals. The average progression rates in PTA (dB/year) in the right and left ears were 0.5 and 0.9, respectively, in one individual with a frameshift variant (family 3: III-1), and were 1.1 and 1.5, respectively, in the other individual with a nonsense variant (family 5: IV-1). However, the audiograms for the two individuals showed a more marked progression in hearing thresholds at the lower and higher frequencies than at the middle frequencies, which were used for the calculation of PTA (Fig 2).

Caloric testing and cVEMP data could be obtained for three unrelated individuals. One of the three individuals with vertigo/dizziness showed normal bilateral vestibular findings. However, the right ear of another individual without vertigo/dizziness (family 2: IV-1) showed decreased caloric response and no cVEMP response, which suggested a dysfunctional lateral semicircular canal and saccule.

### Intervention for HL

Information on the intervention for HL was obtained for 13 individuals (Table 2). Two individuals with bilateral profound HL underwent cochlear implantation, and one individual was scheduled for surgery. Information on postoperative audiometric examinations, including sound field audiometry using calibrated warble tones, and speech audiometry using the Japanese monosyllabic word list (67-S), was available for one individual (family 12: II-6). The average hearing threshold (0.5, 1, 2, and 4 kHz) was 45 dB when wearing the cochlear implant. The maximum speech discrimination score improved from 0% at 100 dB sound pressure level (SPL) prior to implantation to 70% at 70dB SPL with the implant.

## Discussion

MPS has facilitated the detection of causative variants for HL even in small families. Fourteen variants in *POU4F3* have been reported previously (Table 3). Among the 14 variants, seven were detected by targeted MPS technology [10–14, 17, 18]. In the present study, we implemented targeted MPS for a large series of HL patients, and found an additional 12 novel *POU4F3* likely pathogenic variants from 15 unrelated families. The incidence of *POU4F3* variants was 0.6% (15/2,549) among the families with hereditary HL, and 2.5% (15/602) among the families with autosomal dominant HL in the Japanese population. This finding shows that *POU4F3* variants represent the third largest cause of autosomal dominant HL in Japan, following *KCNQ4* variants (6.6%) [36] and *TECTA* variants (2.9%) [37]. Therefore, *POU4F3* is an important deafness gene in autosomal dominant HL patients, particularly in patients with mid- or high-frequency HL.

**Table 3:** Summary of clinical features associated with *POU4F3* variants from previous studies.

				HL		Pure-Tone Audiometry			
Nucleotide Change	Exon	Amino Acid Change	Domain	Onset	Progression	Severity of HL	Audiometric Configuration	Family Origin	Reference
whole deletion of *POU4F3*				11–13 y	Yes	moderate to profound	MF, HF	Brazil	Freitas, 2014
c.120+1G>C	1			0–40 y	Yes	moderate to profound	flat	China	He, 2016
c.337C>T	2	p.Gln113Ter		14–40 y	Yes	moderate to severe	flat, HF	China	Zhang, 2016
c.491C>G	2	p.Pro164Arg		N/A	N/A	mild to profound	flat, HF	China	Wei, 2014
c.602delT	2	p.Leu201fs	POU-specific	16–30 y	Yes	mild to profound	HF	China	Cai, 2016
c.603_604delGG	2	p.Val203fs	POU-specific	N/A	N/A	N/A	N/A	China	Yang, 2013
c.662_675del14	2	p.Gly221fs	POU-specific	20 y	N/A	severe	HF	Korea	Lee, 2010
c.668T>C	2	p.Leu223Pro	POU-specific	4–44 y	Yes	mild to severe	flat, MF, HF	Netherland	Collin, 2008
c.694G>A	2	p.Glu232Lys	POU-specific	20's	N/A	moderate to severe	HF	Korea	Beak, 2012
c.865C>T	2	p.Leu289Phe	Homeobox	13–20 y	Yes	mild to profound	flat, MF, HF	Netherland	Collin, 2008
c.884_891del8	2	p.Ile295fs	Homeobox	18–30 y	Yes	moderate to severe	HF	Israel	Vahava, 1998
c.932T>C	2	p.Leu311Pro	Homeobox	10–20 y	Yes	moderate to profound	HF	China	He, 2016
c.977G>A	2	p.Arg326Lys	Homeobox	10–50's	N/A	mild to moderate	flat, HF	Korea	Kim, 2013
c.1007delC	2	p.Ala336fs		0 y	Yes	moderate to severe	N/A	Japan	Mutai, 2013

Abbreviations: y, year(s) old; HL, hearing loss; HF, high-frequency hearing loss; MF, mid-frequency hearing loss; NC: not classified; N/A, not available

Nine of the previously reported 14 *POU4F3* variants exist within the two DNA-binding domains, the POU-specific and Homeobox domains (Table 3). In the present study, five variants were located in the POU-specific domain, and three variants were located in the Homeobox domain. The other two frameshift variants and a nonsense variant were predicted to result in the absence of the two DNA-binding domains. The two DNA-binding domains play a crucial role in the high-affinity binding DNA of the POU transcription factor. The mechanism by which *POU4F3* variants lead to HL remains unknown. However, functional studies have suggested that *POU4F3* variants within the POU-specific domain (p.Leu223Pro) or Homeobox domain (p.Leu289Phe, and p.Ile295fs) decrease the binding ability to target DNA, reduce activation of reporter gene expression, and /or are partially mislocalized in the cytoplasm [8, 38]. In addition, although heterozygous *Pou4f3* ^+/-^ mice had normal hearing [4], the whole deletion of *POU4F3* has been reported to be responsible for HL in a DFNA15 family [19]. Based on these findings, haploinsufficiency rather than a dominant-negative effect is thought to be the most likely mechanism underlying the HL.

**Fig 3:**
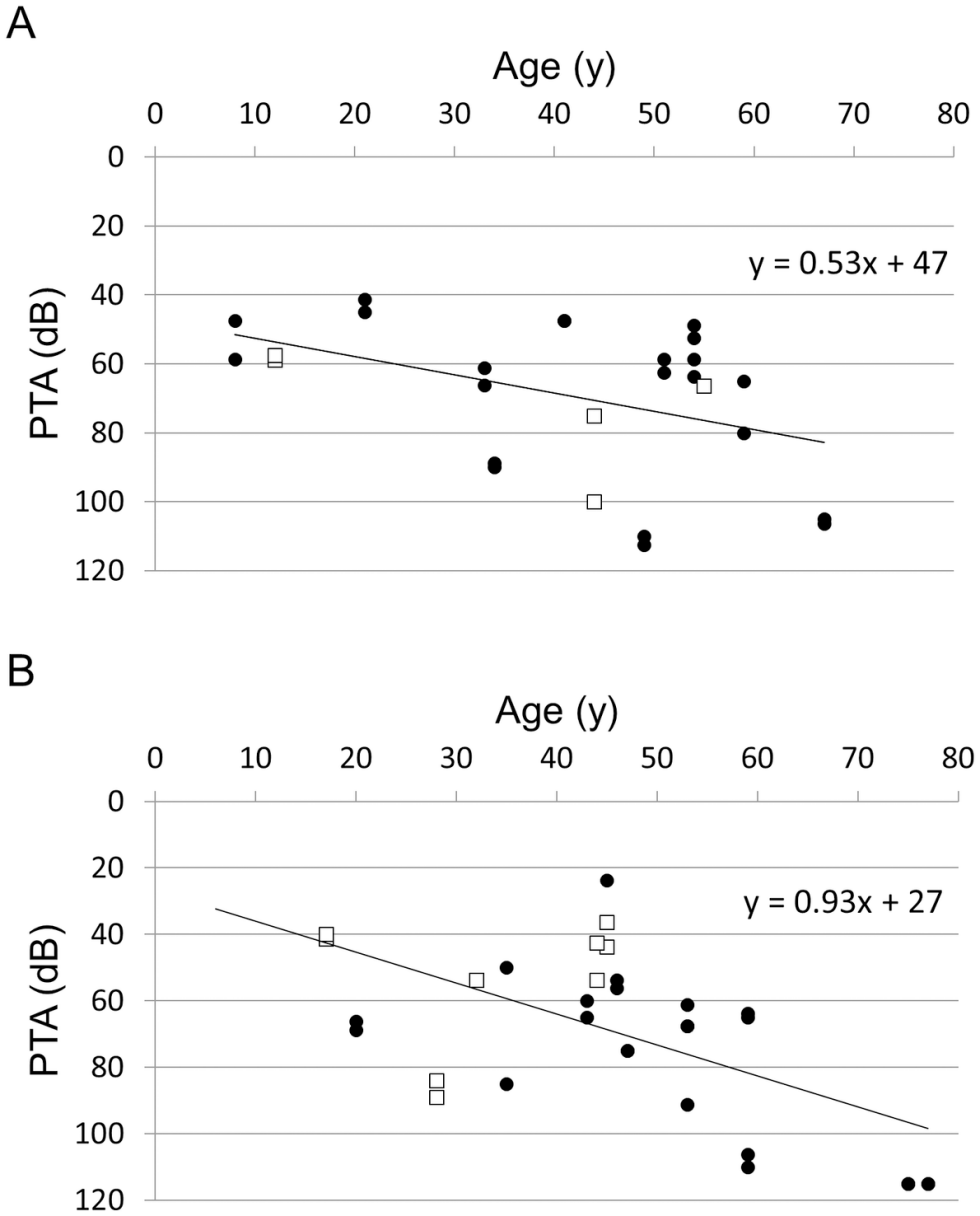
Association between pure-tone average (PTA) and age. The association between age and PTA in the bilateral ears of 15 probands (black circles) in our study and eight probands (white squares) in previously reported DFNA15 families are plotted according to variant type; truncating (nonsense, frameshift variants, or a whole deletion) variants (A) and non-truncation (missense) variants (B).

The onset age and degree of HL in DFNA15 patients have been reported to vary markedly even within the same family [39]. Similarly, the onset and severity of HL in the affected individuals in our study exhibited wide variations. Kim et al. [12] reported that a missense variant in *POU4F3* caused late-onset HL. In order to clarify whether variant type affected the onset of HL, we investigated the relationships between age and PTA according to variant type. The association between age and PTA in the bilateral ears of the 15 probands in our series and of eight probands in previously reported DFNA15 families [3, 8–10, 12, 17, 19] is plotted in Fig 3. When serial audiograms could be obtained for an individual, we used the PTA from the first audiogram and the age at testing. The results suggested that probands with missense variants show later onset and faster progression of HL compared to those with nonsense, frameshift variants, or a whole deletion. Alternatively, a truncating variant may cause earlier onset and slower progression of HL compared to a non-truncating variant.

Previous studies reported that DFNA15 results in progressive HL. Pauw et al. [39] performed a linear regression analysis of cross-sectional audiometric data in a large Dutch family with a p.Leu289Phe variant. The average threshold progression rate (dB/year) was 1.0 at 4 kHz and 1.4 at 8 kHz, which were slightly higher than the approximate rate of 0.8 observed across the frequencies from 0.25 to 2 kHz. In our study, all individuals for whom information could be obtained, complained of progressive HL. Serial audiograms available for two unrelated individuals showed hearing deterioration, especially at the lower and higher frequencies. Overlapping audiograms for each age period showed that hearing at 4 and 8 kHz was predominantly affected with age. Taken together, the progression of HL appears to be relatively slow at the middle frequencies.

In the present study, mid- and high-frequency HL type audiometric configurations were most commonly observed in patients with *POU4F3* variants. This finding is consistent with those in previous studies, as shown in Table 3. However, all affected ears showed mid-frequency HL during the period from 20–39 years of age, whereas some affected ears had high-frequency HL from 40–59 years. Therefore, the audiometric configuration may depend on the age at testing in some ears.

Laterality in HL has not been mentioned in previous studies. However, we found a difference of >10 dB in PTA between the right and left ears in approximately 20% of affected individuals. Asymmetric hearing can be recognized in patients with other types of nonsyndromic hereditary HL, including DFNB4 caused by variants in *SLC26A4* [40], and DFNA9 caused by *COCH* variants [41]. A patient with *SLC26A4* or *COCH* variants can show transient or permanent asymmetric HL because of fluctuating and/or acute deterioration in HL. In the present study, one individual suffered from acute deterioration in hearing during the course of HL. Therefore, acute deterioration of hearing may be one of the causes for asymmetric hearing in DFNA15 patients.

Among a number of nonsyndromic deafness genes, variants in *SLC26A4*, *COCH*, *MYO7A*, *GRHL2*, and *CLIC5* as well as *POU4F3* are known to cause vestibular symptoms and/or dysfunction [42]. In 19 patients with p.Leu289Phe variants in a large Dutch DFNA15 family, eight patients had moderate to severe vestibular symptoms, and 15 patients showed abnormal findings on vestibular-ocular examination and/or caloric testing [43]. In this study, three individuals underwent caloric testing and cVEMP. As a result, one individual exhibited unilateral abnormal findings on both vestibular examinations on the more severe HL side. By contrast, four (17%) out of 23 affected individuals suffered incidences of vertigo/dizziness; however, most individuals did not undergo vestibular examination. We should, therefore, pay more attention to vestibular function in patients with *POU4F3* variants.

We previously reported that genetic screening using MPS is important in patients who are scheduled for cochlear implants or electric acoustic stimulation [44]. In this study, patients who had variants in nonsyndromic deafness genes known to be localized and function in the inner ear showed satisfactory auditory performance, suggesting that the identification of the genetic background was useful in predicting postoperative performance. There have been no previous reports describing cochlear implantation for patients with *POU4F3* variants. In our series, two individuals with bilateral profound HL underwent cochlear implantation. We were able to evaluate postoperative status in one of the two individuals, with the results showing good auditory performance after cochlear implantation. However, more detailed postoperative auditory tests in a larger number of DFNA15 patients are needed to clarify the performance of cochlear implants.

In conclusion, this study using MPS successfully identified 12 novel and likely pathogenic variants, and estimated the incidence of *POU4F3* variants to be 2.5% in Japanese families with autosomal dominant HL. Pure-tone audiograms for our affected individuals showed that the most prevalent configuration was mid-frequency HL type followed by high-frequency HL type, and approximately 20% of the affected individuals showed asymmetry in hearing. Individuals with truncating variants tended to show earlier onset and slower progression of HL compared to those with non-truncating variants, suggesting a possible genotype-phenotype correlation in DFNA15 patients.

## Supporting information

S1 Table 68 genes reported to be causative of hearing loss. (PDF)
