# Supplementary material for: POU4F3 mutation screening in Japanese hearing loss patients: Massively parallel DNA sequencing-based analysis identified novel variants associated with autosomal dominant hearing loss
Source: PLoS One. 2017 May 17;12(5):e0177636. doi: 10.1371/journal.pone.0177636 (PMC5435223; doi:10.1371/journal.pone.0177636)
Supplement: S1 Table — (PDF) [file pone.0177636.s001.pdf]

Supplementary Table S1. 68 deafness-causative genes.

No	Locus Symbol	Gene Symbol	Gene ID	Transcript Variant
1	DFNA1	*DIAPH1*	NM_005219	NM_001079812
2	DFNA2	*KCNQ4*	NM_004700	NM_172163
3	DFNA2	*GJB3*	NM_024009	NM_001005752
4	DFNA3	*GJB6*	NM_006783	NM_001110219
				NM_001110220
				NM_001110221
5	DFNA4	*MYH14*	NM_024729	NM_001145809.
				NM_001077186
6	DFNA5	*DFNA5*	NM_004403	NM_001127454
				NM_001127453
7	DFNA6/14/38	*WFS1*	NM_006005	NM_001145853
8	DFNA8/12/DFNB21	*TECTA*	NM_005422	-
9	DFNA9/31	*COCH*	NM_004086	NM_001135058
10	DFNA10	*EYA4*	NM_004100	NM_172103
				NM_172105
11	DFNA11/DFNB2/USH1B	*MYO7A*	NM_000260	NM_001127179
				NM_001127180
12	DFNA13/DFNB53/STL3	*COL11A2*	NM_080680	NM_080679
				NM_080681
				NM_001163771
13	DFNA15	*POU4F3*	NM_002700	-
14	DFNA17	*MYH9*	NM_002473	-
15	DFNA20/DFNA26	*ACTG1*	NM_001614	NM_001199954
16	DFNA22/DFNB37	*MYO6*	NM_004999	-
17	DFNA25	*SLC17A8*	NM_139319	NM_001145288
18	DFNA28	*GRHL2*	NM_024915	-
19	DFNA36/DFNB7/11	*TMC1*	NM_138691	-
20	DFNA40	*CRYM*	NM_001888	NM_001014444
21	DFNA44	*CCDC50*	NM_178335	NM_174908
22	DFNA48	*MYO1A*	NM_005379	-
23	DFNA50	*MIRN96*		
24	DFNA51	*TJP2*	NM_001170414	NM_004817
				NM_201629
				NM_001170630
				NM_001170415
				NM_001170416

25	DFNA64	*DIABLO*	NM_019887	NM_138929
26		*CEACAM16*	NM_001039213	-
27	DFNB1/DFNA3	*GJB2*	NM_004004	-
28	DFNB3	*MYO15A*	NM_016239	-
29	DFNB4/Pendred	*SLC26A4*	NM_000441	-
30	DFNB6	*TMIE*	NM_147196	-
31	DFNB8/10	*TMPRSS3*	NM_024022	NM_032405
32	DFNB9	*OTOF*	NM_194248	NM_194322
				NM_194323
				NM_004802
33	DFNB12/USH1D	*CDH23*	NM_022124	NM_001171930
				NM_001171931
				NM_001171932
				NM_001171933
				NM_001171934
				NM_001171936
				NM_052836
34	DFNB15/72/95	*GIPC3*	NM_133261	-
35	DFNB16	*STRC*	NM_153700	-
36	DFNB18/USH1C	*USH1C*	NM_153676	NM_005709
37	DFNB22	*OTOA*	NM_144672	NM_001161683
				NM_170664
38	DFNB23/USH1F	*PCDH15*	NM_033056	NM_001142763
				NM_001142764
				NM_001142765
				NM_001142766
				NM_001142767
				NM_001142769
				NM_001142770
				NM_001142771
				NM_001142772
				NM_001142773
39	DFNB24	*RDX*	NM_002906	-
40	DFNB25	*GRXCR1*	NM_001080476	-
41	DFNB28	*TRIOBP*	NM_007032	NM_138632
				NM_001039141

42	DFNB29	*CLDN14*	NM_144492	NM_001146077
				NM_001146078
				NM_001146079
				NM_012130
43	DFNB30	*MYO3A*	NM_017433	-
44	DFNB31/USH2D	*WHRN*	NM_015404	NM_001083885
				NM_001173425
45	DFNB35	*ESRRB*	NM_004452	-
46	DFNB36	*ESPN*	NM_031475	-
47	DFNB39	*HGF*	NM_000601	NM_001010931
				NM_001010932
				NM_001010933
				NM_001010934
48	DFNB42	*ILDR1*	NM_001199799	NM_001199800
				NM_175924
49	DFNB48	*CIB2*	NM_006383	NM_001271888
				NM_001271889
50	DFNB49	*MARVELD2*	NM_001038603	NM_001244734
51	DFNB59	*DFNB59*	NM_001042702	-
52	DFNB61	*SLC26A5*	NM_206883	NM_001167962
				NM_206884
				NM_206885
				NM_198999
53	DFNB63	*LRTOMT*	NM_001145307	NM_001145308
				NM_001205138
				NM_145309
54	DFNB66/67	*LHFPL5*	NM_182548	-
55	DFNB70	*PNPT1*	NM_033109	-
56	DFNB74	*MSRB3*	NM_198080	NM_001031679
				NM_001193460
				NM_001193461
57	DFNB77	*LOXHD1*	NM_144612	NM_001173129
				NM_001145472
				NM_001145473
58	DFNB79	*TPRN*	NM_001128228	-
59	DFNB82	*GPSM2*	NM_013296	-
60	DFNB84	*PTPRQ*	NM_001145026	-
61	DFNB89	*KARS*	NM_005548	NM_001130089

62	DFNB91	*SERPINB6*	NM_004568	NM_001195291
63	DFNB93	*CABP2*	NM_016366	-
64	USH2A	*USH2A*	NM_007123	NM_206933
65	DFNX1	*PRPS1*	NM_002764	NM_001204402
66	DFNX2	*POU3F4*	NM_000307	-
67	DFNX4	*SMPX*	NM_014332	-
68	DFNX6	*COL4A6*	NM_001847	NM_033641
